# Properties of functional brain networks correlate with frequency of psychogenic non-epileptic seizures

**DOI:** 10.3389/fnhum.2012.00335

**Published:** 2012-12-20

**Authors:** Elham Barzegaran, Amir Joudaki, Mahdi Jalili, Andrea O. Rossetti, Richard S. Frackowiak, Maria G. Knyazeva

**Affiliations:** ^1^Department of Computer Engineering, Sharif University of TechnologyTehran, Iran; ^2^Département des Neurosciences Cliniques, Centre Hospitalier Universitaire Vaudois, University of LausanneLausanne, Switzerland; ^3^Laboratoire de Recherche en Neuroimagerie, Département des Neurosciences Cliniques, Centre Hospitalier Universitaire Vaudois, University of LausanneLausanne, Switzerland; ^4^Départment de Radiologie, Centre Hospitalier Universitaire Vaudois and University of LausanneLausanne, Switzerland

**Keywords:** EEG, functional connectivity, graph theory, cross-correlation analysis, clustering co-efficient, modularity, resilience

## Abstract

Abnormalities in the topology of brain networks may be an important feature and etiological factor for psychogenic non-epileptic seizures (PNES). To explore this possibility, we applied a graph theoretical approach to functional networks based on resting state EEGs from 13 PNES patients and 13 age- and gender-matched controls. The networks were extracted from Laplacian-transformed time-series by a cross-correlation method. PNES patients showed close to normal local and global connectivity and small-world structure, estimated with clustering coefficient, modularity, global efficiency, and small-worldness (SW) metrics, respectively. Yet the number of PNES attacks per month correlated with a weakness of local connectedness and a skewed balance between local and global connectedness quantified with SW, all in EEG alpha band. In beta band, patients demonstrated above-normal resiliency, measured with assortativity coefficient, which also correlated with the frequency of PNES attacks. This interictal EEG phenotype may help improve differentiation between PNES and epilepsy. The results also suggest that local connectivity could be a target for therapeutic interventions in PNES. Selective modulation (strengthening) of local connectivity might improve the skewed balance between local and global connectivity and so prevent PNES events.

## Introduction

Psychogenic non-epileptic seizures (PNES) are involuntary paroxysmal events that, in contrast to epileptic seizures, are unaccompanied by epileptiform EEG discharges but related to a broad spectrum of psychologically traumatic life events from child maltreatment or neglect to participation in armed conflicts (Briere and Elliott, 2003; Reuber, 2009; Salinsky et al., 2011). The facts that such events are considerably more frequent in population at large than PNES suggests a specific cerebral basis for PNES predisposition that we set out to discover. Many attempts to show specific PNES associated brain features have failed to identify them conclusively (Reuber, 2008). Indeed, Reuber states: In conclusion, I cannot really answer the question whether PNES are an expression of “neurologic” pathology. The current evidence suggests that PNES do not occur without “psychogenic,” constitutional or developmental factors, however, “neurologic” factors can play an important etiologic role.”

However, recent research engenders cautious optimism about revealing objective brain features in patients with PNES. LaFrance et al. reported that PNES patients have decreased plasma levels of brain-derived neurotrophic factor (BDNF), which is involved in regulating synaptic reorganization throughout life (LaFrance et al., 2010). Using voxel-based morphometry Labate et al. found cortical atrophy in the motor and premotor cortices of the right hemisphere and in the cerebellum bilaterally (Labate et al., 2012). van der Kruijs et al. showed altered functional connectivity in the brain networks of PNES patients based on resting-state functional MRI (van der Kruijs et al., 2012).

Having hypothesized that abnormality of functional cerebral networks may predispose to PNES (Knyazeva et al., 2011b) we set out to test this suggestion. We characterized cerebral functional connectivity in PNES patients by means of the whole-head surface topography of multivariate phase synchronization (MPS) in interictal resting-state EEGs. Although this measure of intra-regional synchronization showed no significant differences between PNES patients and matched controls, its values within the prefrontal and parietal cortices inversely correlated with individual PNES frequency. As commented in the accompanying editorial (Duncan, 2011) this study, being a pilot by nature, raised the intriguing question whether EEG synchrony could be a marker of brain dysfunction in PNES.

One way to answer this question is to comprehensively characterize brain networks in this condition. Techniques from graph theory allow such an analysis. These have been increasingly applied to model functional and structural networks in the normal brain and under various pathological conditions [for recent reviews see Bassett and Bullmore (2009); Bullmore and Sporns (2012)]. In principle, graph theory considers large-scale brain networks, comprising functionally or anatomically distinct regions and inter-regional pathways that exhibit specific non-random patterns with properties such as small-worldness (SW) and scale-free degree distribution (Bullmore and Sporns, 2009, 2012). Graph theoretical analysis reveals the economical small-world structure of brain networks. These are characterized by much higher local connectivity (i.e., clustering coefficient) than random graphs, while sharing similar global connectivity (i.e., average path length or efficiency) with them (Bullmore and Sporns, 2012). Brain functional networks are cost-efficient in the sense that they provide efficient parallel processing for low connection costs. Brain disorders disrupt anatomical and functional brain networks. Abnormalities of brain networks have been shown in schizophrenia (Lynall et al., 2010; Jalili and Knyazeva, 2011), in Alzheimer's disease (Stam et al., 2009; Tahaei et al., 2012), and in epilepsy (Horstmann et al., 2010; Liao et al., 2010). To the best of our knowledge, general topological properties of brain networks have never been studied in what are considered predominantly psychogenic conditions such as PNES.

Here we report the results of graph theoretical modeling of EEG-based functional networks in PNES patients compared to healthy matched controls.

## Methods

### Patients and control subjects

Thirteen patients with PNES and 13 age- and sex-matched controls were enrolled in the study and provided informed consent in accordance with the guidelines of the local Ethics Committee of the University of Lausanne. The analysis of whole-head topography of power spectral density and of MPS in resting-state EEG in this population was recently reported (Knyazeva et al., 2011b). All the instrumental procedures conformed to the Declaration of Helsinki (1964) of the World Medical Association concerning human experimentation. PNES was diagnosed according to (Benbadis et al., 2004). Briefly, following clinical suspicion, each subject underwent prolonged inpatient or outpatient video-EEG recording lasting up to 24 h, including a spontaneous or induced “seizure episode” (by means of verbal suggestion, hyperventilation, intermittent photic stimulation, or NaCl injection in the presence of an experienced neurologist). The episode was considered diagnostic only if it resembled a typical event for each subject, and in the formal absence of any argument in favor of an epileptic origin (including a detailed history taken by an experienced epileptologist (Andrea O. Rossetti) and normal brain imaging, EEG recording, clinical examination, and blood chemistry). None of the PNES patients had epileptic seizures.

The demographic, clinical, and pharmacological data for all patients are presented in Table 1. All subjects were right-handed (median age 35.9 years; standard deviation 14.6 years; range 18–61; 62% women). The median period between manifestation with PNES and diagnosis was two months, ranging from 1 day to 20 years. Most patients (77%) had convulsive episodes. PNES frequency was assessed by patient reports; in subjects with short-lasting illnesses, duration data were extrapolated using the total number of episodes as the numerator and disease duration as the denominator: the median frequency was eight per month (with a wide range of less than 1 per week to 50 per day). While 31% of subjects had no pharmacological treatment at the time of EEG, roughly half of them (46%) were taking benzodiazepines, and the remainder was on antiepileptic agents and/or antidepressant medications. Thirteen healthy controls were selected from our EEG database based on age and sex matching (median age 36.1 years; standard deviation 14.4; range 18–61 years; 62% women). They were recruited from a database of students of the University of Lausanne and CHUV employees for projects in cognitive neuroscience (Knyazeva et al., 2006, 2011a). The control subjects had neither a history of neurological or psychiatric problems, nor head injury with loss of consciousness. The EEGs of these volunteers were recorded in the rest-eyes-closed condition using the same equipment as for the patient group (see hereafter).

**Table 1:** **Clinical characteristics of PNES patients**.

**Patient #**	**Sex**	**Age (decade)**	**Symptom duration to EEG**	**Number of episodes/month**	**Loss of contact**	**Convulsions**	**Treatment**
1	M	6th	1day	300	yes	no	sertraline
2	F	4th	3 years	1	yes	yes	lamotrigine, citalopram, olanzapine, zolpidem
3	F	2nd	1 day	30	yes	yes	no
4	M	2nd	2 years	8	yes	yes	lorazepam
5	M	3rd	1 month	16	yes	yes	clonazepam
6	M	4th	5 days	90	yes	no	no
7	F	5th	10 years	1	yes	no	alprazolam
8	F	6th	20 years	4	yes	yes	lamotrigine, clonazepam, clobazam, alprazolam, lorazepam, venlafaxine
9	F	6th	1 year	1500	no	yes	diazepam, midazolam
10	M	3rd	5 years	0.1	yes	yes	no
11	F	5th	2 months	4	no	yes	no
12	F	4th	10 months	8	no	yes	Sertraline, clorazepate
13	F	2nd	7 days	30	yes	yes	lorazepam

EEG stands for electroencephalogram, F for female, and M for male.

### EEG recording and processing

Interictal resting EEGs for this analysis were collected within a few days of diagnosis with a 128-channel Geodesic Sensor Net (EGI, OR, USA) for 3–4 min. Subjects were seated with eyes closed. All electrode impedances were kept under 30 kΩ (the recommended limit for the high-input-impedance EGI amplifiers is 50 kΩ). Because of low signal to noise ratio, 17 sensors from the outer ring of the sensor net were not considered, which left 111 sensors for further analysis. The recordings were made with a vertex reference using a low-pass filter set to 100 Hz. The signals were digitized at 500 Hz with a 12-bit analog-to-digital converter; they were filtered (FIR, band-pass of 1–50 Hz) and re-referenced against the common average reference (CAR). The interpretation of surface CAR EEG is limited because of contamination by volume conduction and reference electrode effects (Nunez et al., 1997). These unwanted effects were minimized with the high resolution Laplacian transformed EEG signals, which isolates source activity under each sensor (Srinivasan et al., 2007). For computing Laplacian transform of EEG signals, we used the CSD toolbox (psychophysiology.cpmc.columbia.edu/Software/CSDtoolbox).

To obtain greater confidence in the correlation estimates, signals were segmented into non-overlapping 1-s epochs. Using short segments for analysis allowed us to accumulate 156 ± 61 artefact-free epochs in patients and 164 ± 54 in controls. Artifacts in all channels were edited off-line; first automatically, based on an absolute voltage (100 μV) and a transition threshold (50 μV), and then on the basis of a thorough visual inspection. Sensors recording artifactual EEG signals (>20% of the recording time) were corrected using the bad channel replacement tool (NS 4.2 EGI, USA). Further EEGs were filtered to obtain time series in the bands of interest including delta (1–3 Hz), theta (3–7 Hz), alpha (7–13 Hz), and beta (13–30 Hz) frequency bands.

### Constructing brain functional networks

**Figure 1:**
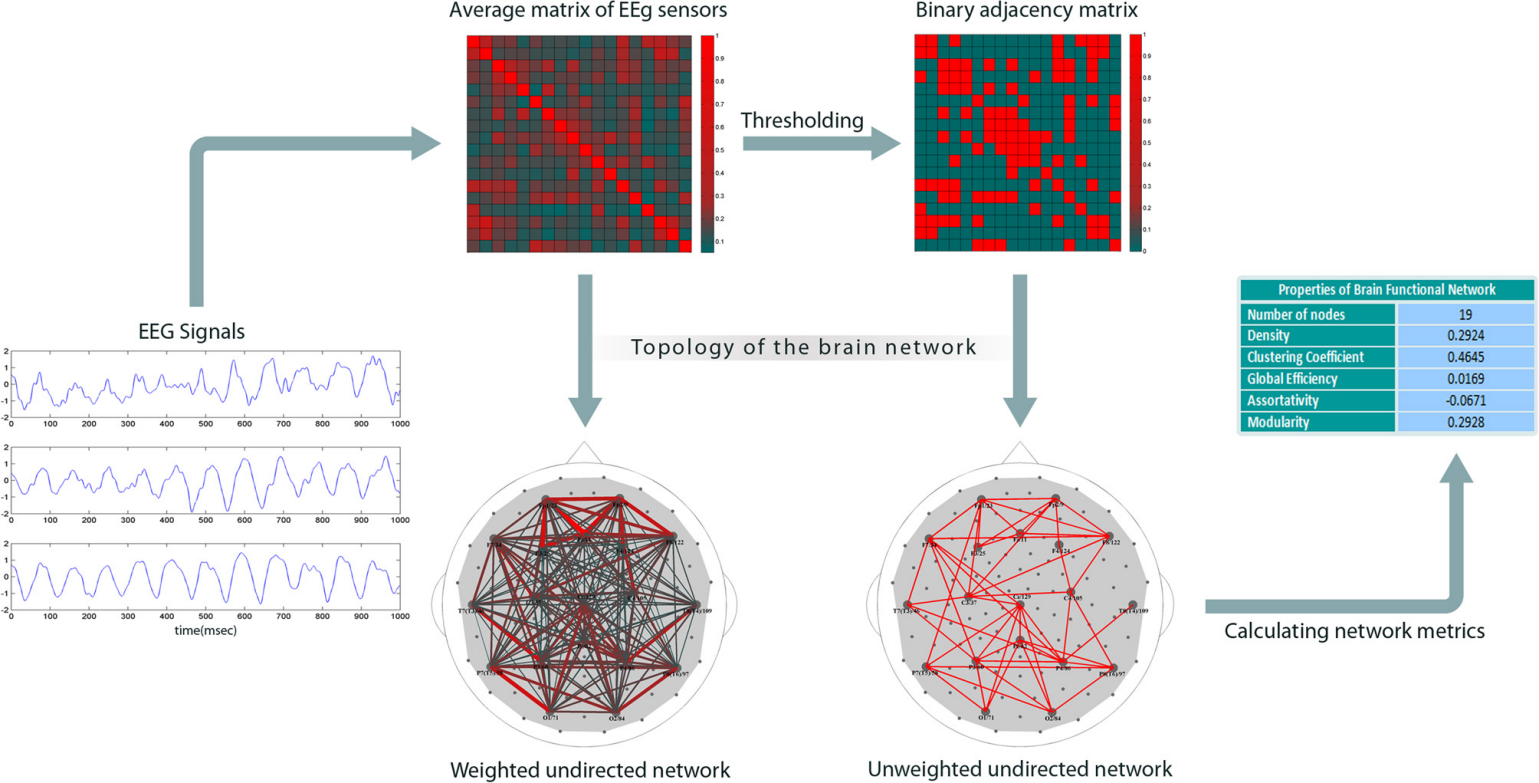
**Construction of brain networks from EEG signals.** The plots on the left show sample EEGs taken over a time period of one second. The next step is to compute pair-wise correlations to obtain a weighted cross-correlation matrix (rows and columns represent the nodes). Then, the matrix is reduced to a binary form by comparing each entry with a threshold (the threshold is set such that the network has a specific density); the links with correlation values less than the threshold are set to 0; others to 1. Finally, graph theoretical metrics are calculated for the binary network.

Figure 1 shows our method for constructing EEG-based functional brain networks. For each subject, the network metrics were calculated epoch-wise, and then averaged over all the available epochs, and, finally, these individually typical values were used for the network analysis.

Since we considered each electrode as a node, correlation analysis, suggested for extracting network topology (Zalesky et al., 2012), resulted here in weighted 111 × 111 correlation matrices. The next step is to build binary connectivity matrices out of the weighted correlation matrices. The method for constructing binary networks is to threshold the weighted correlation matrices; if the correlation between two nodes is larger than a certain threshold, the corresponding entry in the binary adjacency matrix is set to 1, otherwise to 0. Networks can be defined arbitrarily on the basis of different thresholds, but may not be comparable. Indeed, binarizing two correlation matrices based on a specific threshold value might result in two networks with different density, i.e., number of links. An alternative approach is to build binary networks based on network density, that is, to threshold them in such a way that they have equal density values (Achard and Bullmore, 2007; Tahaei et al., 2012).

**Figure A1:**
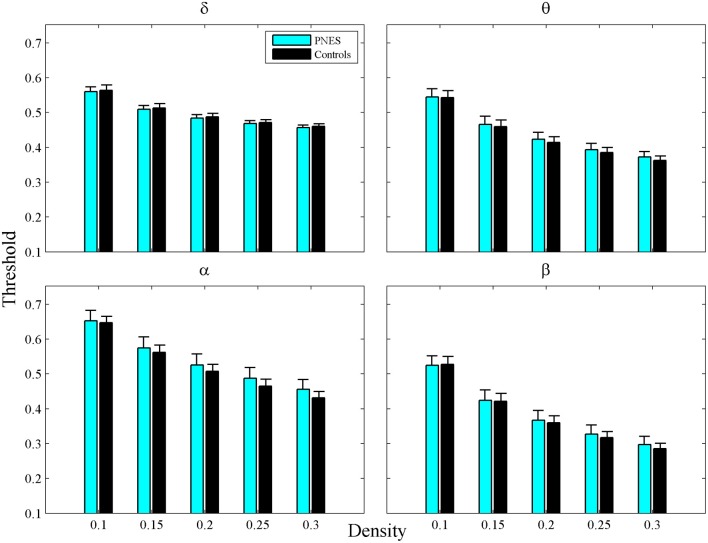
**Thresholds as a function of network density for PNES patients and controls.** The plots show cross-correlation values used to obtain a network at each density by thresholding a subject's weighted correlation matrix. Other designations are as Figure 2.

For a network of size *N*, the network density, defined as the number of links divided by *N*(*N* − 1)/2, shows how dense its connections are. In order to build the binary adjacency matrices, we found the thresholds, which did not differ significantly between groups for any correlation matrix and density value, (Figure A1). The networks constructed in this way have the same density, i.e., the same number of links. First, we used the 0.05–0.3 density range binned at 0.002 intervals for constructing the individual binarized networks for each density value. Second, a number of neurobiologically meaningful graph theory metrics were computed based on these binary networks. Finally, each metric was averaged over five density ranges (0.05–0.1, 0.1–0.15, 0.15–0.2, 0.2–0.25, and 0.25–0.3) for each subject and EEG frequency band. The resulting estimates served as input values for statistical assessments.

### Graph theory metrics

During network extraction, a number of global and local graph theory measures were computed. Degree of nodes is the simplest graph theory metric, which gives valuable information on its properties. Degree is defined as a total number of neighbors that the node has. Nodes with high degrees are called hubs. Here we defined hubs as nodes with a significantly higher degree than the average degree of the network at *P* < 0.05, Wilcoxon's ranksum test. Hubs have essential role in information propagation through the network, its resiliency and functionality.

Metrics such as clustering coefficient and modularity determine functional segregation in the brain, which refers to its ability to locally process information in parallel processing streams. The clustering coefficient (*C*)—a metric quantifying the local connections in the network—qualifies the presence of loops of order three and is calculated here by counting all triangular connections existing in the graph and dividing that number by all the theoretically possible triangular connections (Watts and Strogatz, 1998). Clustering co-efficient of node *i* (*C*_*i*_) is calculated as

(1) $$C_{i}=\frac{1}{N}\frac{\sum_{k,j}a_{kj}a_{ki}a_{ji}}{k_{i}(k_{i}-1)},$$

where *N* is the network size, *a*_*ij*_ is the corresponding element of the adjacency matrix between nodes *i* and *j*, and *k*_*i*_ is the degree of node *i* that is obtained by summing all incoming links to *i*. The clustering coefficient of the network is obtained by averaging over those of individual nodes, as

(2) $$C=\frac{1}{N}\sum_{i}C_{i},$$

Many real-world networks including those of the brain show modular structure. The following index has been proposed to calculate the degree of modularity in a network (Girvan and Newman, 2002)

(3) $$Q=\sum_{m}\text{​}\left[e_{ii}-{\left(\sum_{j\in m}e_{ij}\right)}^{2}\right]\text{​},$$

where *m* is the predetermined number of non-overlapping modules, and *e*_*ij*_ equals the proportion of all links connecting nodes in module *i* with those in module *j*. *Q* quantifies the degree of modular structure with *m* modules, in which the larger the *Q* the more modular the network. To calculate the modularity index, we used a bottom-up hierarchal algorithm (Newman, 2004).

Network efficiency (*E*) is another important metric indicating the ability of a network to integrate information (Rubinov and Sporns, 2010). Global efficiency of a network is calculated as (Latora and Marchiori, 2001)

(4) $$E=\frac{1}{N(N-1)}\sum_{i,j}\frac{1}{l_{i,j}}.$$

It has been shown that many real-world networks including brain networks have small-world topology—a structure that is neither pure random, nor regular, but somewhere in between (Watts and Strogatz, 1998; Buzsaki, 2006). To capture this property, SW metric, which estimates clustering coefficient (*C*) and average path length (*L*)—which is obtained by averaging the *l*_*i*, *j*_'s—of a given network compared to those of a random network having the same number of nodes and edges (*C*_random_, *L*_random_) has been proposed (Humphries and Gurney, 2008):

(5) $$\text{SW}=\frac{\frac{C}{L}}{\frac{C_{\text{random}}}{L_{\text{random}}}},$$

Network resiliency (*r*) is linked to its degree-degree correlation (Newman, 2002, 2003). The latter can be estimated by the assortativity measure as follows (Newman, 2002)

(6) $$r=\frac{\frac{1}{T}\sum_{j\;>\;i}k_{i}k_{j}a_{ij}}{\frac{1}{T}\sum_{j\;>\;i}\left(k_{i}^{2}+k_{j}^{2}\right)a_{ij}-{\left[\frac{1}{T}\sum_{j\;>\;i}\frac{1}{2}(k_{i}+k_{j})a_{ij}\right]}^{2}},$$

where *T* is the number of the links in the network, *a*_*ij*_ is the corresponding element of the adjacency matrix between nodes *i* and *j*, and *k*_*i*_ is the degree of node *i*. When *r* > 0, a network is assortative, i.e., likely to contain mutually coupled hub nodes, whereas *r* < 0 implies a disassortative network, for which hub nodes of high degree are unlikely to be inter-linked.

### Statistical assessment

The statistical analysis was based on the averaged individual graph theoretical measures (see “Constructing brain functional networks,” for details), which were computed by means of the brain connectivity toolbox (sites.google.com/a/brain-connectivity-toolbox.net/bct/Home). The differences between the patients and controls were considered significant at *P* < 0.05 (Wilcoxon's ranksum test). To account for multiple comparisons, the *P*-values were corrected with Benjamini-Hochberg (BH) false discovery rate method (Benjamini and Hochberg, 1995). All the statistical assessments were performed using the Statistics toolbox in MatLab.

### Correlations with clinical data

In order to investigate the links between clinical features of PNES and graph theory metrics, we calculated the Spearman correlations coefficient (*CC*) of network metrics with the average number of episodes that patients experienced a month and considered them significant at *P* < 0.05 (Student's *t*-test; BH corrected). The Spearman correlation technique calculates the relationship between two variables using their rankings, and, thus, is not sensitive to outliers. Due to this advantage, it is useful for the data sets with a high variability, as the frequency of PNES episodes in our case.

## Results

**Figure 2:**
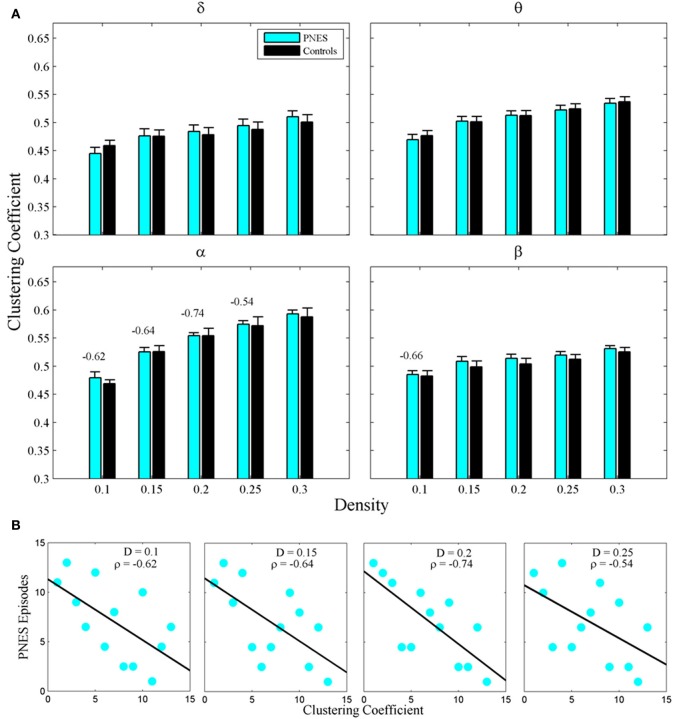
**Clustering co-efficient as a function of network density for PNES patients and controls. (A)** The plots show mean values and standard errors for clustering co-efficients of functional brain networks as a function of density in the groups of PNES patients (cyan bars) and controls (black bars) for different frequency bands. For each density value *d*, the clustering co-efficient is averaged over the range (*d* − 0.05, *d*), e.g., the values for density 0.1 are averaged over the density range 0.05−0.1 (see “Methods” for details). Values above the lines indicate the significant correlation (Spearman correlation; *P* < 0.05; BH corrected) between the number of episodes per month in PNES and clustering co-efficient of their brain functional network. **(B)** Scatterplots for correlations between clustering co-efficients and the number of PNES episodes per month obtained for EEG alpha band.

**Figure 3:**
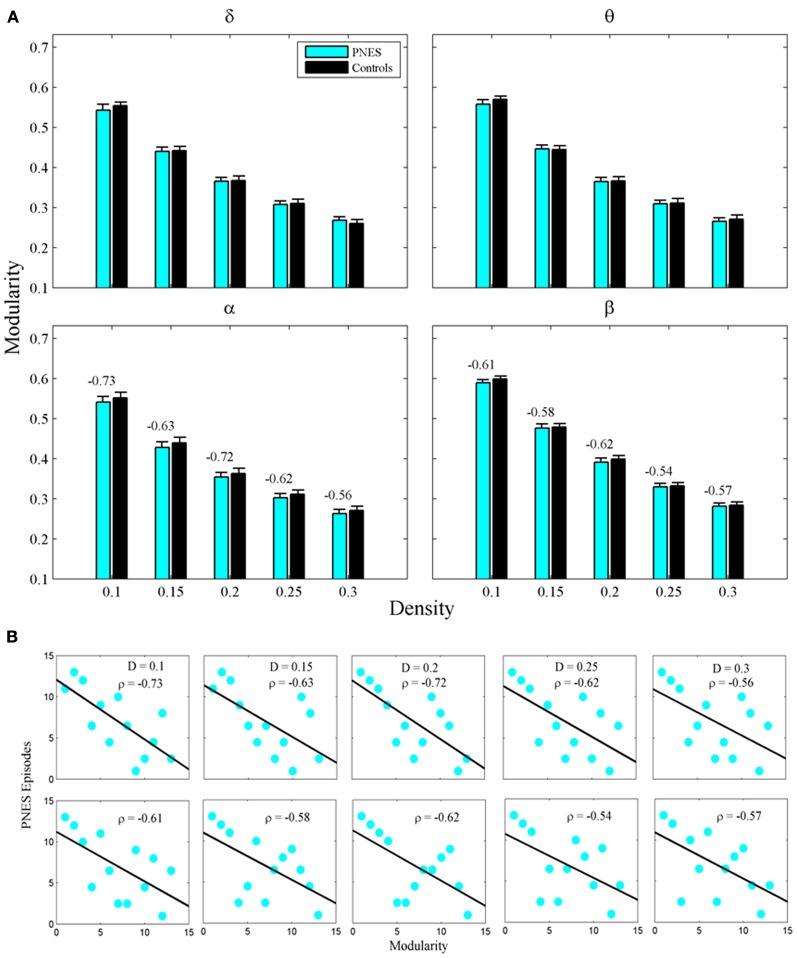
**Modularity index as a function of network density for PNES patients and controls. (A)** The plots show the modularity of functional brain networks as a function of density in PNES patients and controls. **(B)** Scatterplots for correlations between modularity metric and the number of PNES episodes per month obtained for EEG alpha band (upper row) and beta band (lower row). Other designations are as Figure 2.

Figure 2A shows the clustering coefficient as a function of network density across delta-beta EEG frequency bands. The clustering coefficient relates to local connectivity and is likely to increase with network density, as can be seen in our results. We found no systematic significant differences for this metric in PNES patients vs. controls. Yet the clustering co-efficient showed inverse correlations with the frequency of PNES episodes for broad range of density values in alpha band (Figure 2B; mean correlation co-efficient (*CC*) = −0.64, *P* < 0.05; BH corrected) and for low density in beta band (*CC* = −0.66, *P* < 0.05; BH corrected). These negative correlations indicate that frequent attacks are associated with poor local (intraregional) connectivity in the cortical networks. Similarly, another metric of local connectivity, the modularity, showed no significant differences between PNES patients and controls (Figure 3A), but inversely correlated with PNES frequency in alpha (Figure 3B; mean *CC* = −0.65, *P* < 0.05, BH corrected) and beta bands (mean *CC* = −0.58, *P* < 0.05, BH corrected).

**Figure 4:**
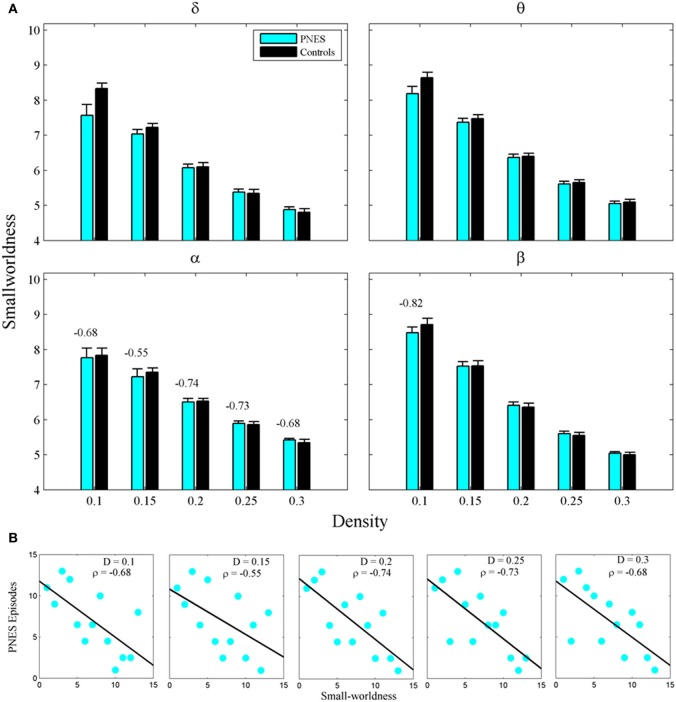
**Small-worldness as a function of network density for PNES patients and controls. (A)** The plots show the small-worldness index of functional brain networks as a function of density in PNES patients and controls. **(B)** Scatterplots for correlations between small-worldness metric and the number of PNES episodes per month obtained for EEG alpha band (upper panels) and beta band (lower panels). Other designations are as Figure 2.

The widely used index of SW also failed to differentiate PNES patients from controls (Figure 4A), however, in alpha band, this metric showed significant inverse correlations with PNES frequency for all densities (Figure 4B; mean *CC* = −0.68, *P* < 0.05; BH corrected) and, in beta band, for low-density networks (*CC* = −0.82, *P* < 0.05; BH corrected). Inverse correlations suggest that the lower the SW of network structure in PNES, the more frequent the psychogenic episodes.

**Figure 5:**
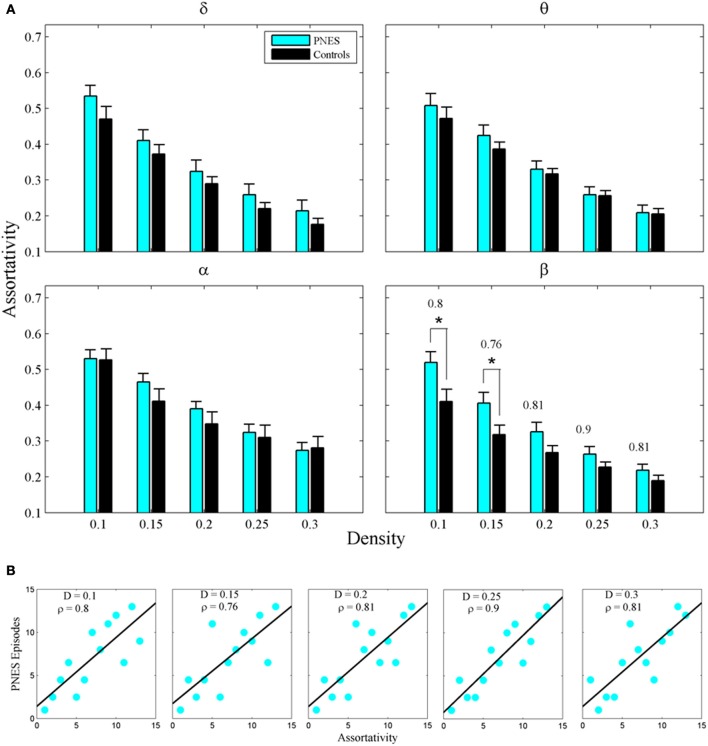
**Assortativity as a function of network density for PNES patients and controls. (A)** The plots show the assortativity of functional brain networks as a function of density in PNES patients and controls. Asterisks indicate that PNES clustering co-efficient is significantly different from that of controls (*P* < 0.05, Wilcoxon's ranksum test; BH corrected). **(B)** Scatterplots for correlations between assortativity metric and the number of PNES episodes per month obtained for EEG alpha band. Other designations are as Figure 2.

**Figure A2:**
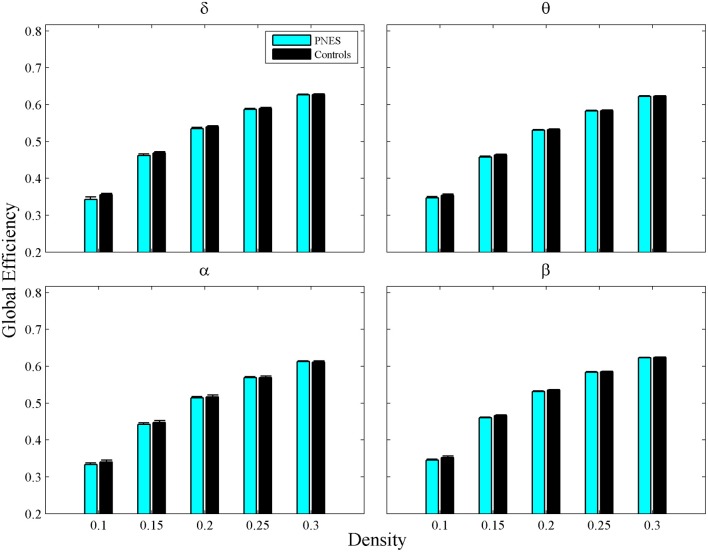
**Global Efficiency as a function of network density for PNES and controls.** The plots show the global efficiency of functional brain networks as a function of density in PNES patients and controls. Other designations are as Figure 2.

The assortativity metric differentiated PNES patients from controls for low densities in the EEG beta band (Figure 5A). Notably, the assortativity values turned out to be higher in PNES patients. This index directly correlated with the number of episodes per month in the beta band (mean *CC* = 0.82, *P* < 0.05; BH corrected) meaning that, in patients, the more assortative functional networks are more prone to the PNES attacks (Figure 5B). Global efficiency revealed neither differences between controls and PNES patients nor significant correlation with the number of episodes per month in the latter group (Figure A2).

**Figure 6:**
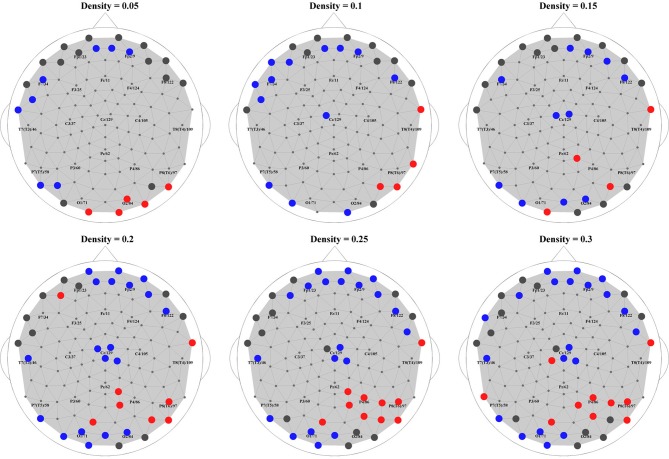
**Hubs in PNES and controls.** The picture shows hub nodes in EEG alpha band. Large dark gray circles show the hub that are common in PNES patients and controls, while red circles show the hubs in PNES patients that are absent in controls, and blue circles, hubs that are manifested only by controls. The networks were binarized at density values of 0.05, 0.1, 0.15, 0.20, 0.25, and 0.30.

Finally, to put the general network properties of PNES patients and controls in the framework of brain topography, we show the distribution of their hub nodes across the density range (Figure 6). Consistently with the findings described above, a clear picture emerged in alpha band (shown in the figure). PNES condition affected the nodes in prefrontal and posterior areas differently: patients lost their prefrontal and left-posterior hubness, while the number of right-hemisphere posterior hubs increased, especially for higher-density networks.

## Discussion

Patients with PNES suffer from sudden occurrences of abnormal behavior, which evokes a question as to the properties of brain dynamic networks that might increase a threshold for physiological interactions or lower it for pathological ones. Although previous empirical studies of PNES loosely used the term “networks” in the context of their building blocks (Labate et al., 2012) or functional connectivity (Knyazeva et al., 2011b; van der Kruijs et al., 2012), it should be emphasized that complex brain networks are more than a collection of building blocks or pair-wise interactions in that they develop new properties that can be best understood within the framework of graph theoretical analysis (Bullmore and Sporns, 2009; Rubinov and Sporns, 2010). This is the first attempt to apply graph theory to a characterization of functional brain networks in PNES. With this approach, we have found that deviant features of EEG-based brain network topology are associated with the occurrence of PNES attacks.

Clustering coefficient—a measure of the local connectedness of a network—did not differ between groups. Inverse correlations with the frequency of PNES episodes show that the lower the local connectivity, the more frequent the PNES attacks. Another statistic characterizing local connectivity—the modularity index—estimates the extent to which a modular structure, i.e., the presence of node clusters with a maximum within-cluster and minimum between-cluster connectivity, is inherent in a network. PNES patients showed close to normal modularity; however, modularity index in the alpha band inversely correlated with PNES frequency. For compatibility with other studies, we also tested SW as a widely used metric, although it is not independent from the measures of local and global connectivity (see Graph theory metrics). Significant inverse correlations of PNES frequency with SW in alpha band are similar to those obtained for clustering coefficient and suggest the effect of local connectivity that biases the balance between segregation and integration allowed by the small-world structure of the brain networks (Micheloyannis et al., 2006; Stam et al., 2006; Bassett and Bullmore, 2009; Lynall et al., 2010). Therefore, based on interictal EEG, these measures revealed that the deficits in local connectivity and/or a skewed balance between local and global connectivity correlate with the frequency of PNES episodes.

These features of local brain connectivity across the whole brain supplement and generalize our recent findings (Knyazeva et al., 2011b). In that study, functional connectivity was estimated via the surface topography of MPS of interictal high-density (Laplacian) EEG in PNES patients vs. controls. The MPS mapping of *intraregional* (i.e., relatively short-distance) synchronization showed widespread inverse correlations between individual frequencies of PNES attacks and MPS in prefrontal and parietal cortices. Moreover, the comparison between synchronization landscapes in the patients receiving benzodiazepines and the patients without this medication showed only patchy, non-uniform differences that could explain neither the topography nor the sign of wide-spread correlations (Knyazeva et al., 2011b). Finally, the hub topography complement this picture by demonstrating in PNES patients the reduced number of hubs in the prefrontal regions against their increased number in the posterior areas. Combining the results from both studies, it is reasonable to conclude that an abnormality of local connectivity in PNES is a factor that contributes to the occurrence of attacks. Intraregional *prefrontal* connectivity appears to be especially critical for the behavioral manifestations characteristic of PNES.

The assortativity metric differentiated PNES patients from controls for low densities in beta band and correlated directly with the frequency of PNES episodes. Assortativity is a measure of brain network resilience (Newman, 2002, 2003). Networks with positive assortativity have a relatively resilient core of mutually interconnected high-degree nodes (hubs). Following Achard et al. (2006), such architecture is a feature of healthy brain organization because of the associated robustness to local cortical hub damage (Achard et al., 2006). The decreased resilience observed in some pathological conditions is interpreted as a loss of network functionality (Liu et al., 2011). In the absence of the examples of increased resilience in other than PNES pathological conditions, it is not a readily interpretable finding. Given that network assortativity directly correlates with the frequency of PNES episodes (the higher the assortativity (i.e., resilience), the higher the number of PNES attacks), it may manifest excessive network rigidity leading to decreased plasticity and capacity for functional reorganization in PNES subjects—an empirical question that merits independent investigation.

The differential diagnosis of PNES represents an important challenge because of the similarity between PNES episodes and epileptic seizures (Dworetzky et al., 2006; Devinsky et al., 2011), and of the considerable therapeutic implications (Razvi et al., 2012). The diagnostic gold standard for PNES is video-EEG. The confirmation of PNES requires typical episodes to be recorded in the absence of epileptiform activity. Yet, a normal EEG does not rule out epilepsy, since in certain cases epileptic seizures do occur in the absence of EEG evidence. The situation is further complicated by an increased prevalence of epilepsy in PNES patients (Reuber et al., 2002; Devinsky et al., 2011). To confirm the diagnosis of PNES, currently, the patient's neurological and psychiatric histories are determinant. Our finding that the topology of interictal functional networks in PNES may predispose to or facilitate PNES episodes suggests that EEG has the potential to further diminish the risk of misdiagnosis beyond a mere demonstration of the absence or presence of visually detectable epileptic features. However, such a change in practice will require confirmation of our results in a much larger patient cohort.

Indeed, modeling studies show that the network properties affect their dynamic behavior. In the hippocampal model, numerical simulations on excitatory neurons coupled through networks with small-world topology revealed that certain changes in the topology cause transitions from normal to seizing and bursting behaviors (Netoff et al., 2004). In the hierarchical modeled networks, their modular structure might present a threshold for system-wide synchronization, and epileptic seizures can arise from the failure of normal modular structure (Kaiser and Hilgetag, 2010). The network analysis of real EEG, fMRI, etc. also suggests that in epilepsy interictal functional networks have clinically relevant topological features. In patients with temporal lobe epilepsy, within the temporal lobe, functional connectivity, clustering coefficient, and the small-world index negatively correlate with disease duration in the broad frequency range of interictal electrocorticogram (van Dellen et al., 2009). In chronic patients with localization-related epilepsy, both local and global efficiency in fMRI-based functional networks are disrupted (Vlooswijk et al., 2011). Therefore, a direct comparison between the architecture of interictal functional networks in PNES vs. epilepsy seems to be a useful step for understanding which features of network patterns are specifically associated with each of the two conditions.

## Conclusion

We have applied graph theory to measure general topological indices of pertinence to complex brain networks in order to characterize functional connectivity in the brains of people with a tendency to PNES episodes. Based on correlations between graph theory metrics and the frequency of PNES attacks, we conclude that, in EEG alpha band, weakness of local connectedness and skewed balance between local and global connectedness predispose to and/or facilitate the occurrence of PNES episodes. Above-normal resiliency in EEG beta band, also correlating with the frequency of PNES attacks, may be interpreted as an excessive rigidity of networks, another feature that makes the attacks possible. This interictal EEG phenotype may help improve differentiation between PNES and epilepsy. The results also suggest that local connectivity could be a target for therapeutic interventions in PNES. Selective modulation (strengthening) of local connectivity might improve the skewed balance between local and global connectivity and so prevent PNES events.

However, because of the heterogeneity and relatively small size of the studied patients' cohort, our findings require further validation. Future longitudinal studies will need to address the relevance of cerebral network topology to the PNES condition. The results will clarify whether such network features are trait- or state-dependent and whether they change with appropriate therapeutic interventions.

### Conflict of interest statement

The authors declare that the research was conducted in the absence of any commercial or financial relationships that could be construed as a potential conflict of interest.
